# Supplementary figures and images for: Modeling cell biological features of meiotic chromosome pairing to study interlock resolution
Source: PLoS Comput Biol. 2022 Jun 13;18(6):e1010252. doi: 10.1371/journal.pcbi.1010252 (PMC9232156; doi:10.1371/journal.pcbi.1010252)

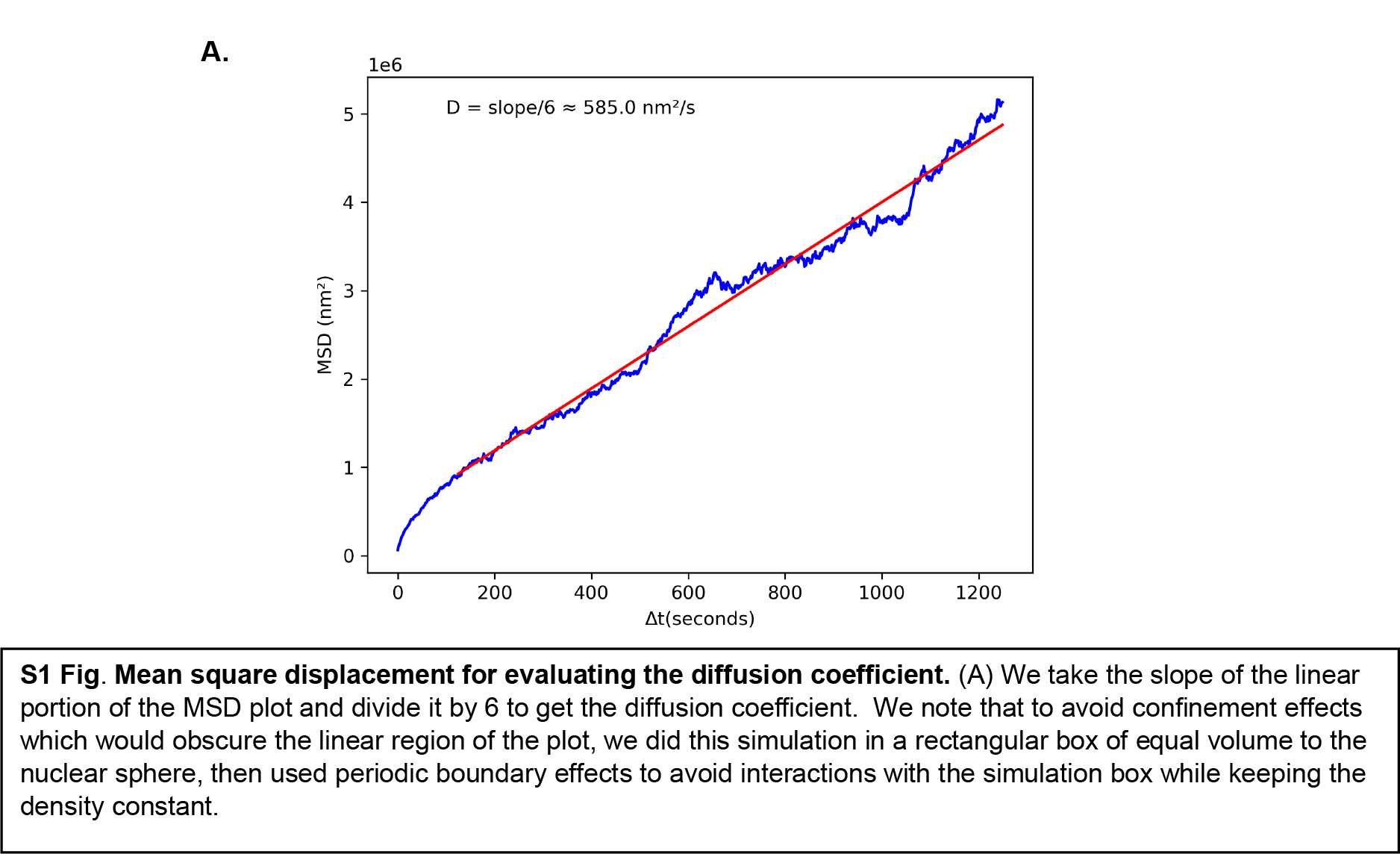

Supplement: S1 Fig — (A) We take the slope of the linear portion of the MSD plot and divide it by 6 to get the diffusion coefficient. We note that to avoid confinement effects which would obscure the linear region of the plot, we did this simulation in a rectangular box of equal volume to the nuclear sphere, then used periodic boundary effects to avoid interactions with the simulation box while keeping the density constant. (TIF) [file pcbi.1010252.s001.tif]

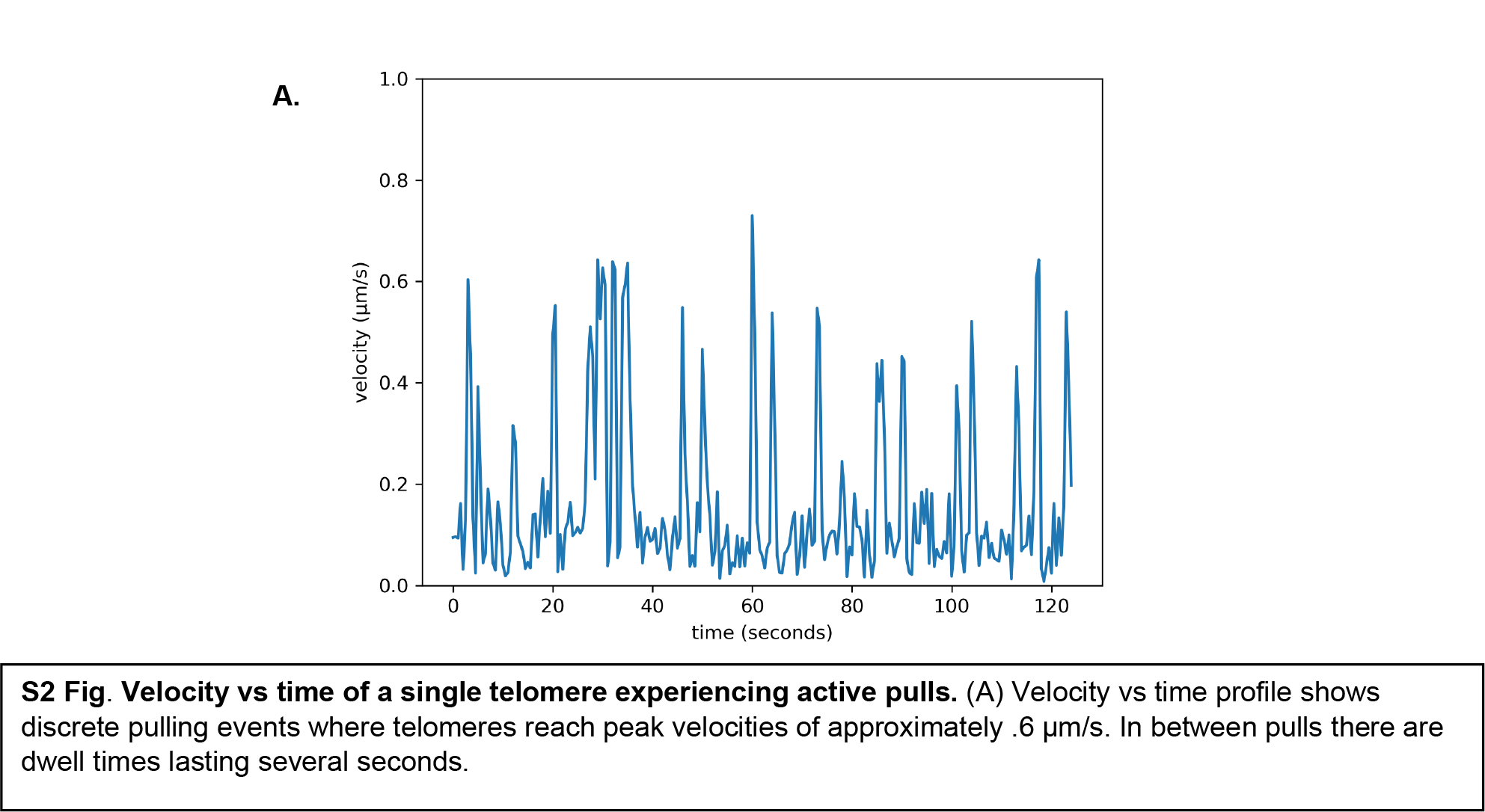

Supplement: S2 Fig — (A) Velocity vs time profile shows discrete pulling events where telomeres reach peak velocities of approximately .6 μm/s. In between pulls there are dwell times lasting several seconds. (TIF) [file pcbi.1010252.s002.tif]

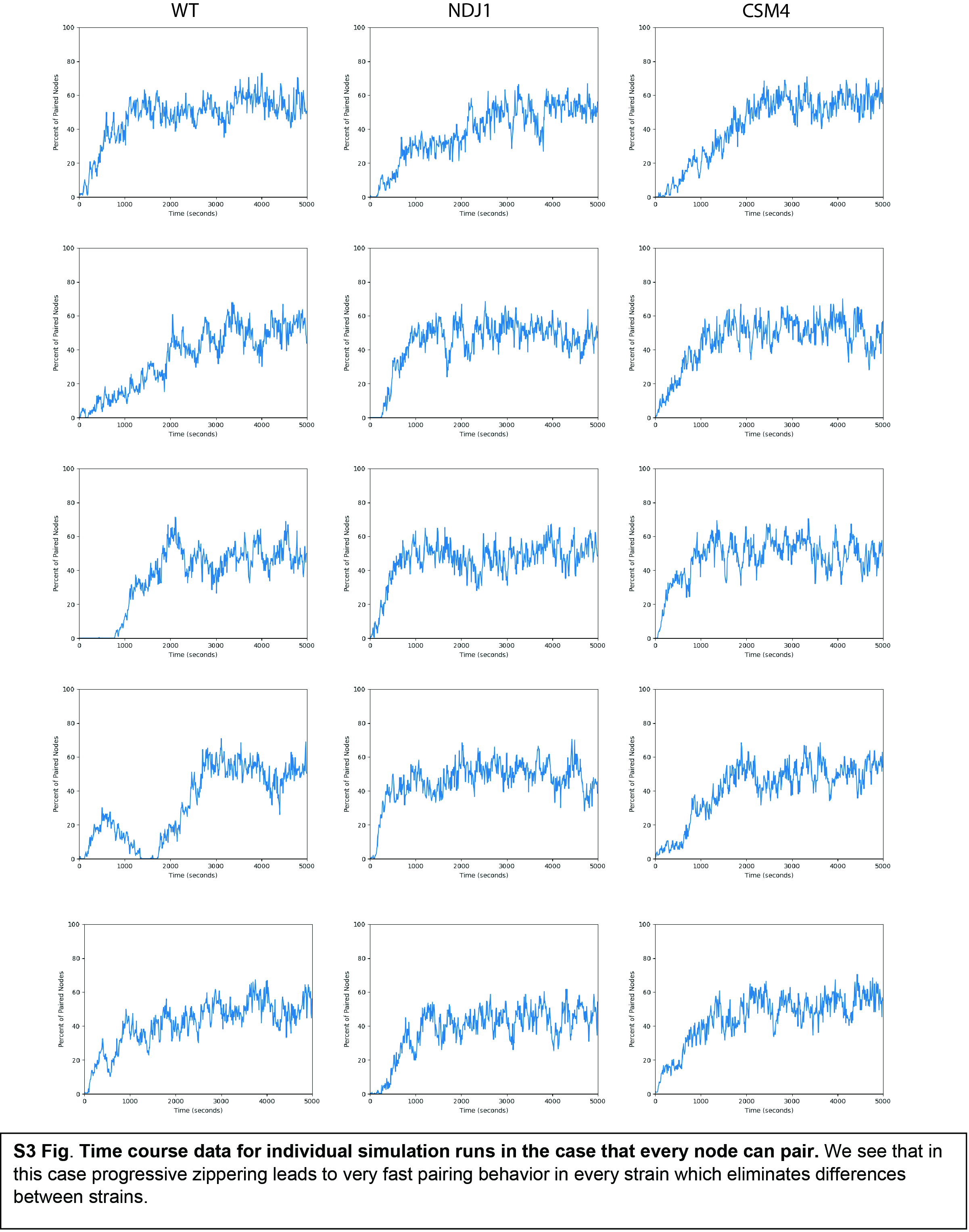

Supplement: S3 Fig — Progressive zippering leads to very fast pairing behavior in every strain which eliminates differences between strains. (TIF) [file pcbi.1010252.s003.tif]

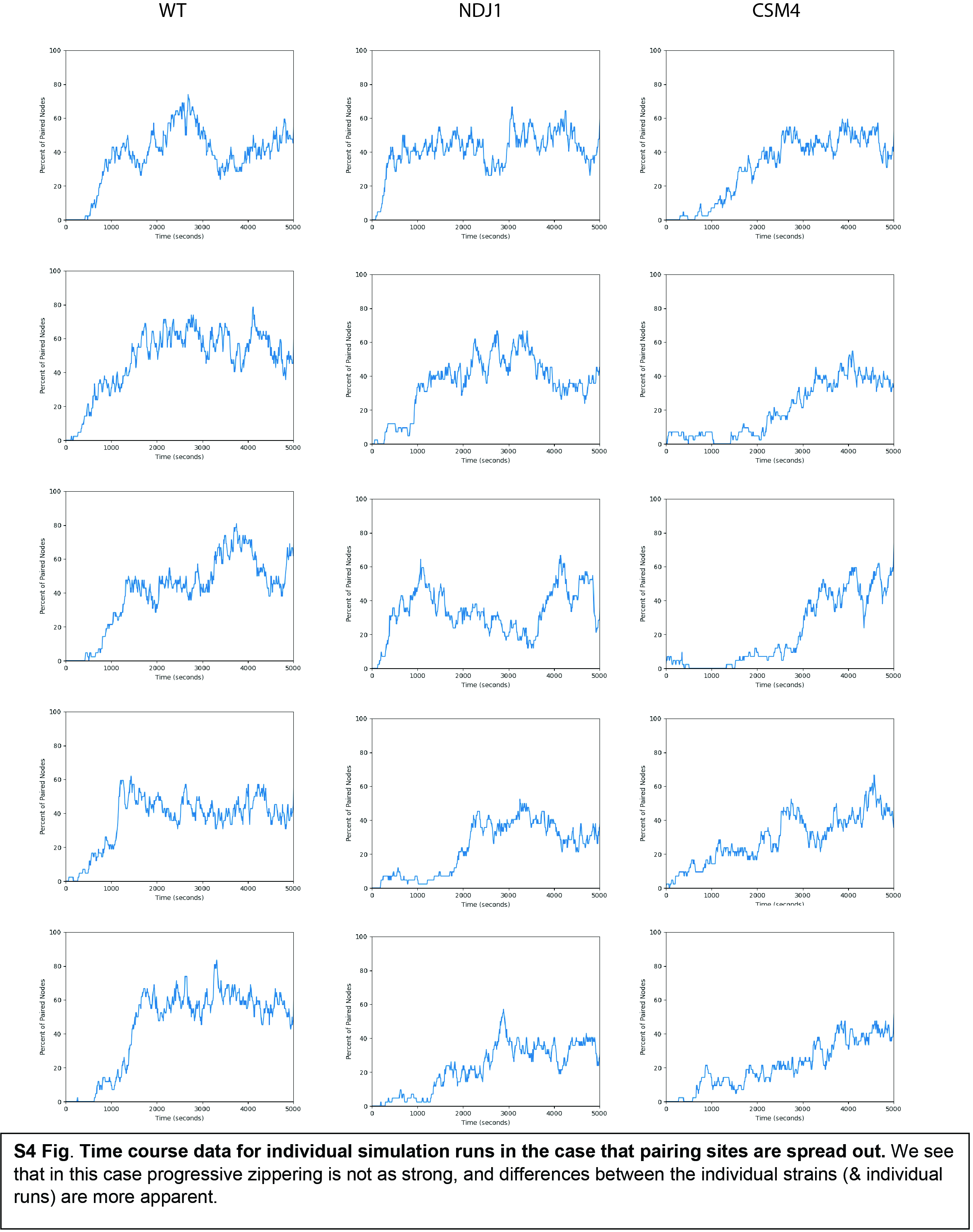

Supplement: S4 Fig — Progressive zippering is not as strong, and differences between the individual strains (& individual runs) are more apparent. (TIF) [file pcbi.1010252.s004.tif]

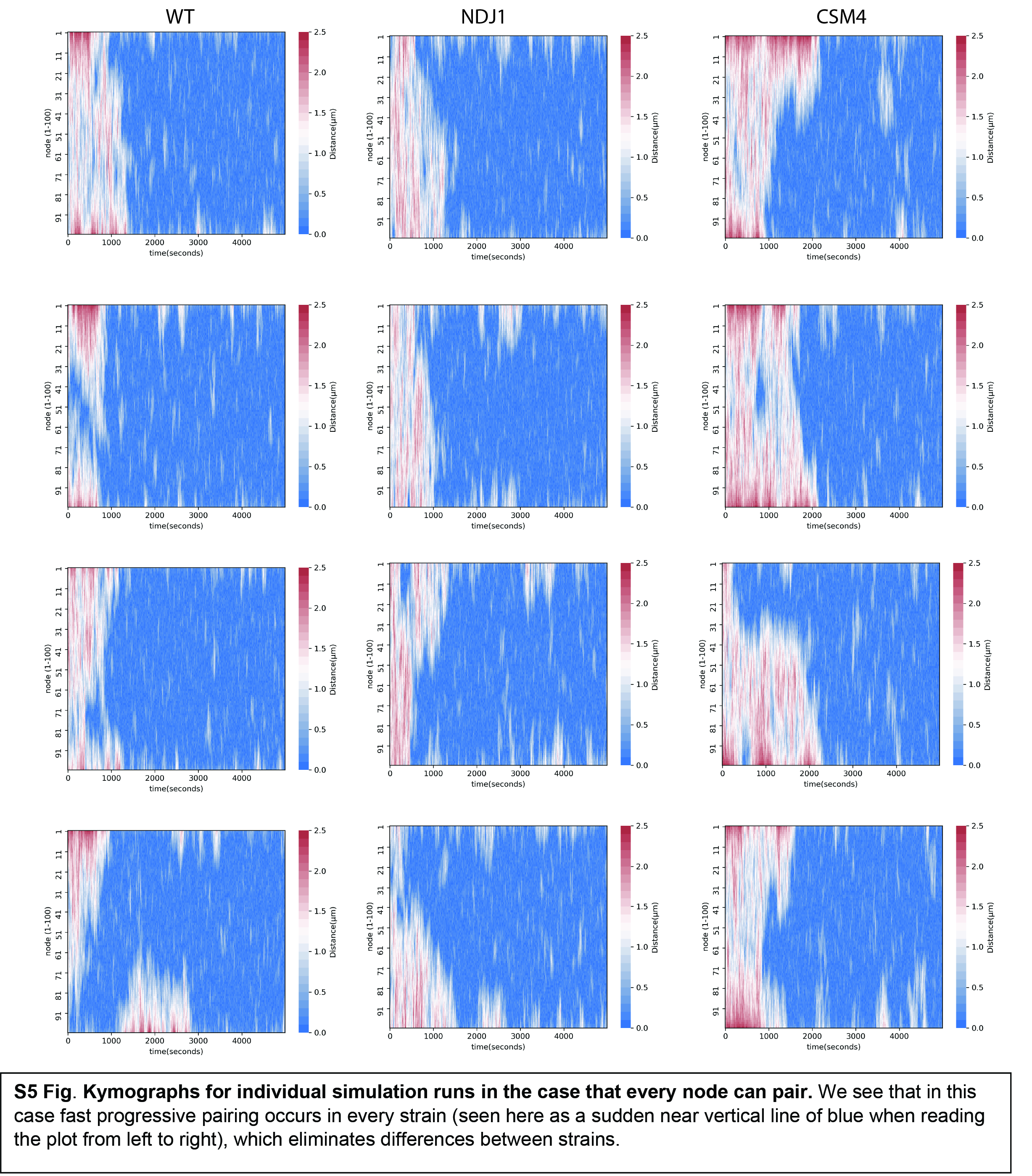

Supplement: S5 Fig — Fast progressive occurs in every strain (seen here as a sudden near vertical line of blue when reading the plot from left to right), which eliminates differences between strains. (TIF) [file pcbi.1010252.s005.tif]

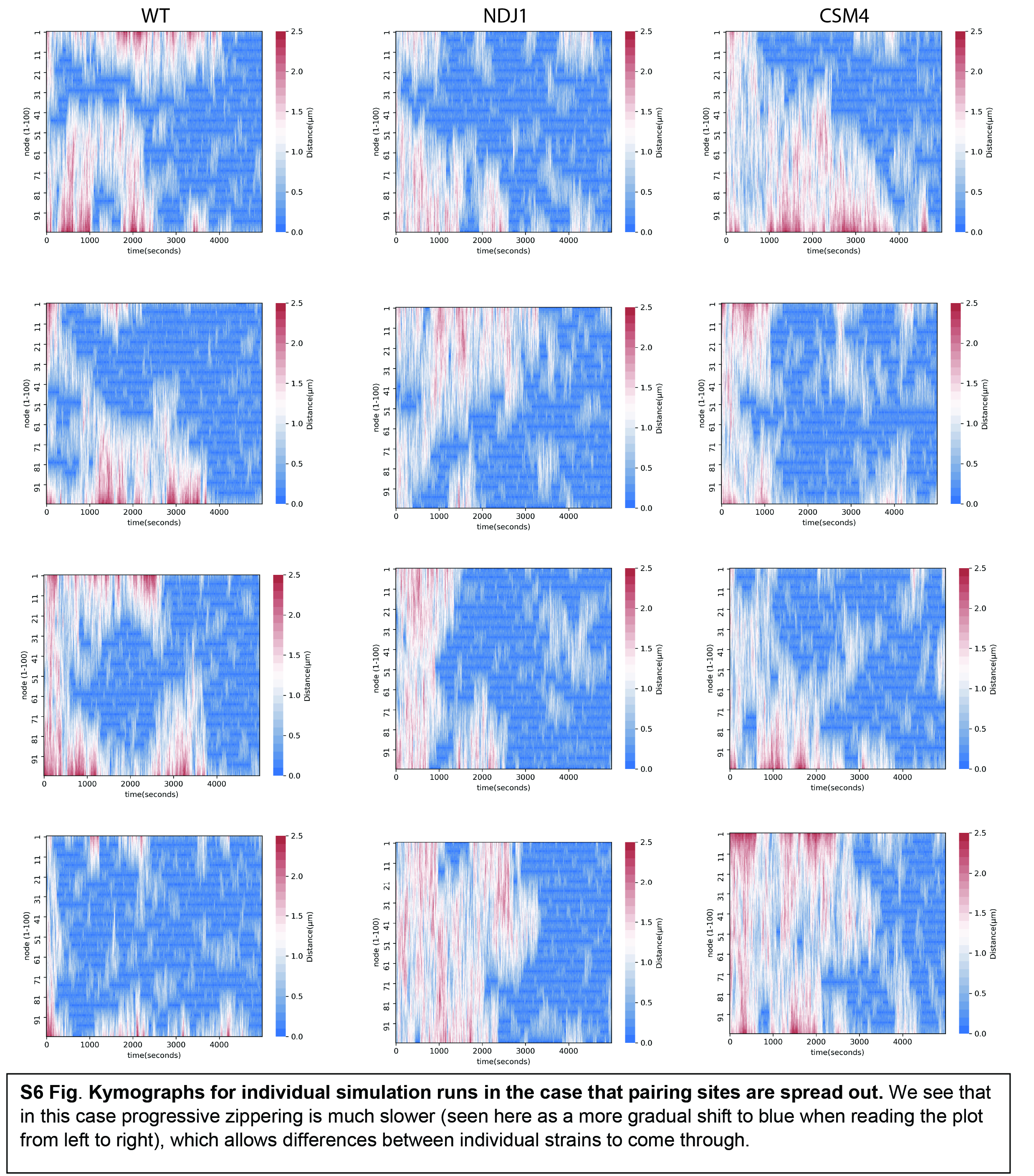

Supplement: S6 Fig — Progressive zippering is much slower (seen here as a more gradual shift to blue when reading the plot from left to right), which allows differences between individual strains to be more apparent. (TIF) [file pcbi.1010252.s006.tif]

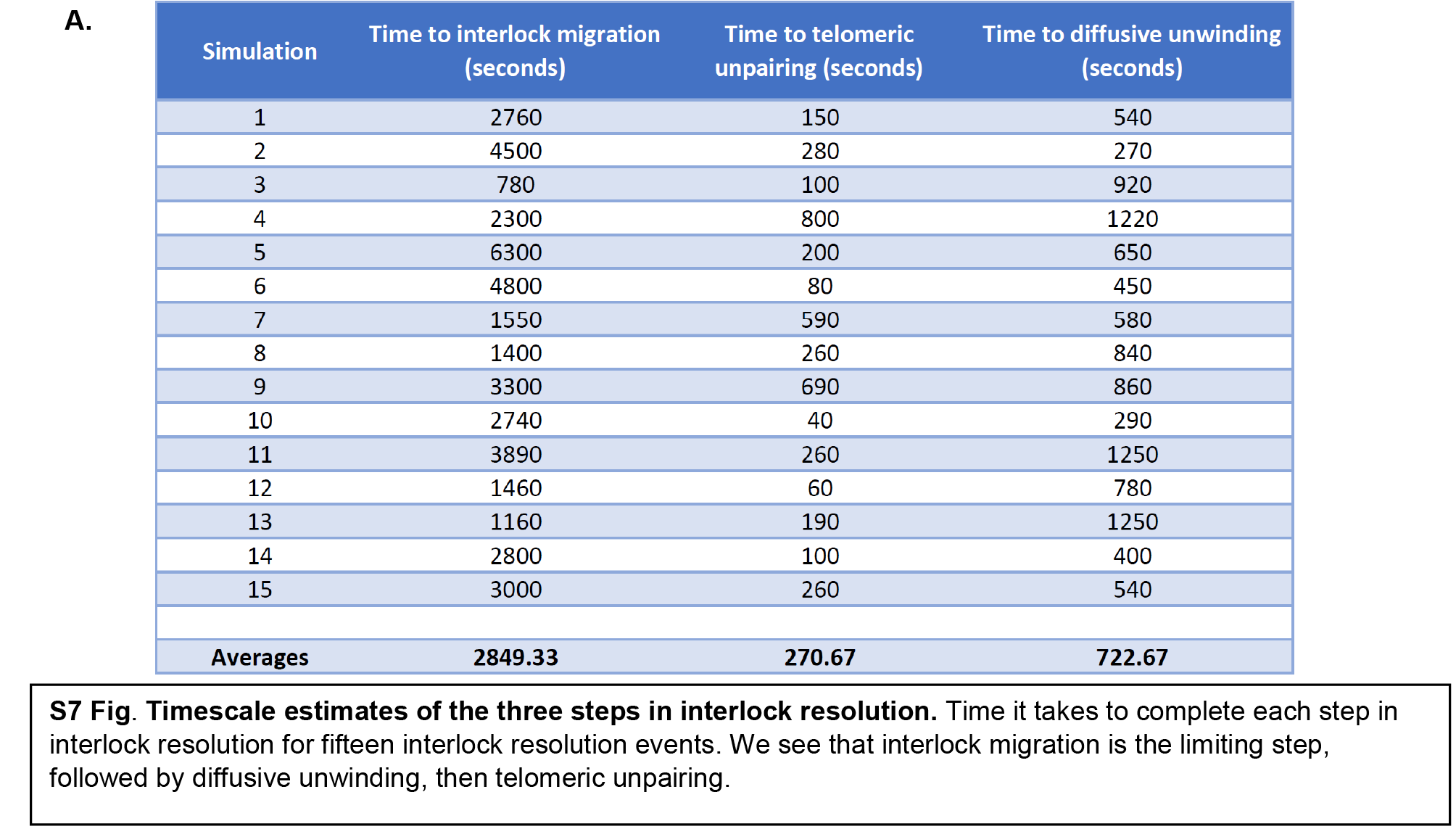

Supplement: S7 Fig — Time it takes to complete each step in interlock resolution for fifteen interlock resolution events. We see that interlock migration is the limiting step, followed by diffusive unwinding, then telomeric unpairing. (TIF) [file pcbi.1010252.s007.tif]

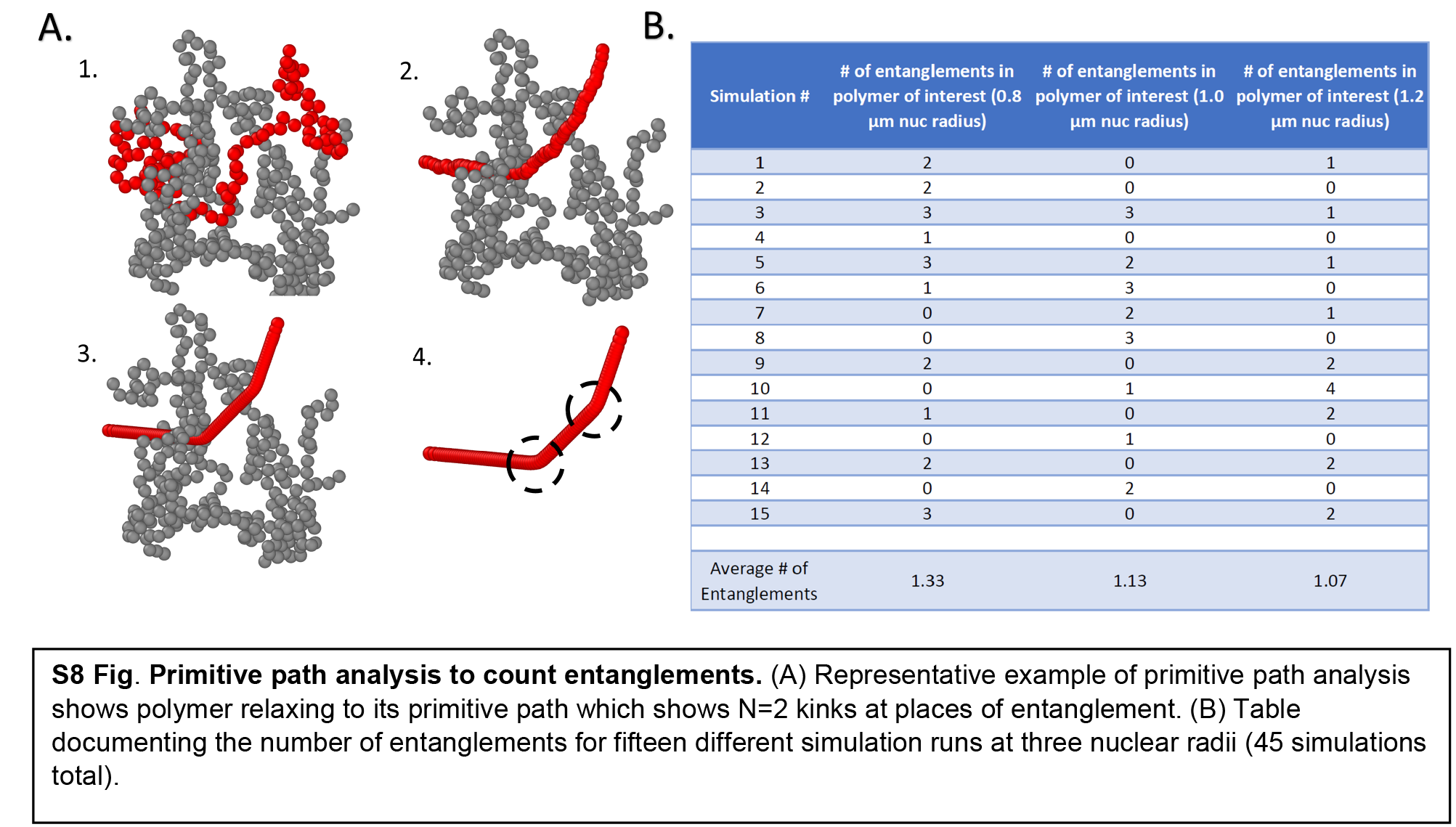

Supplement: S8 Fig — (A) Representative example of primitive path analysis shows polymer relaxing to its primitive path which shows N = 2 kinks at places of entanglement. (B) Table documenting the number of entanglements for fifteen different simulation runs at three nuclear radii (45 simulations total). (TIF) [file pcbi.1010252.s008.tif]

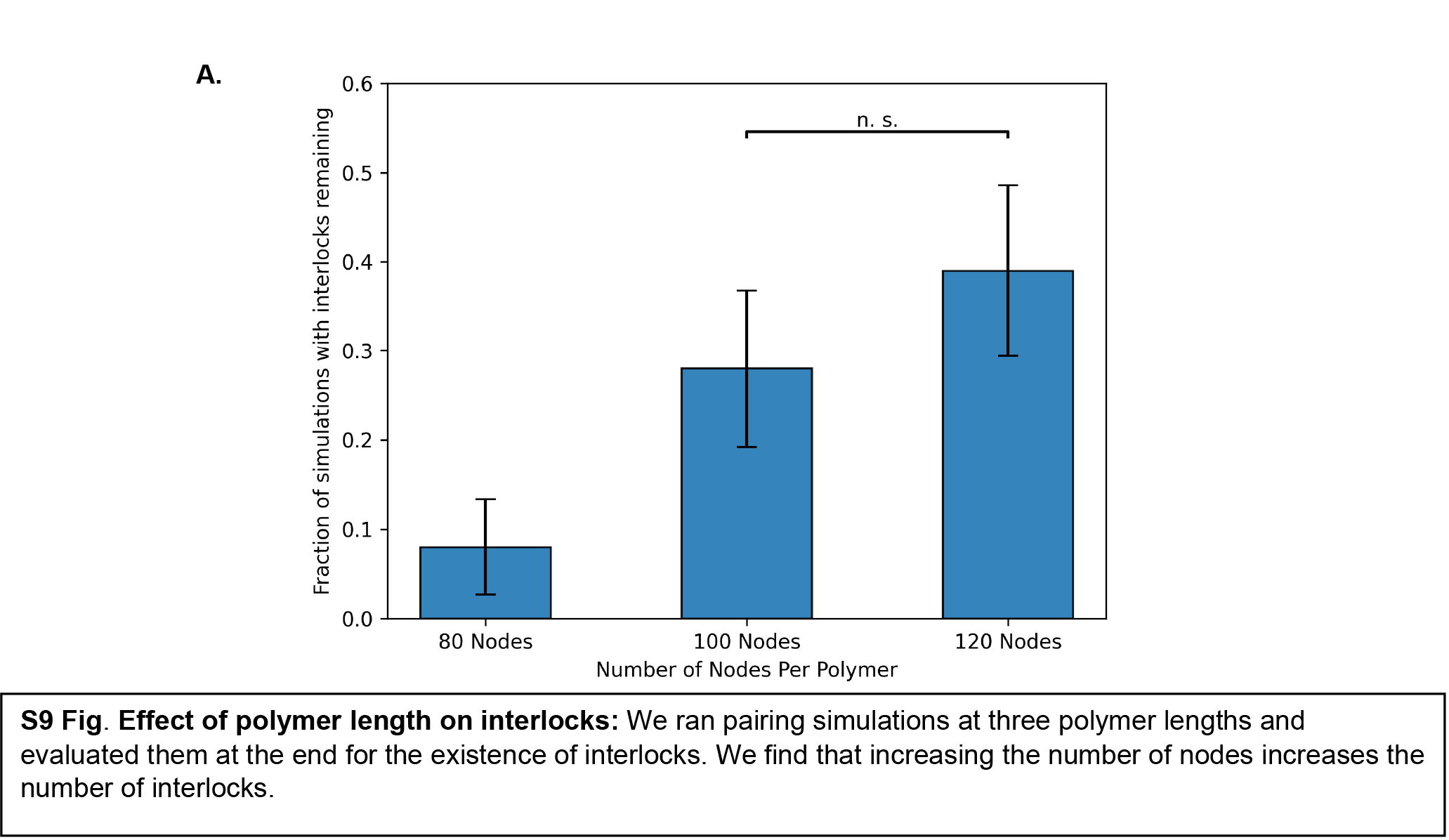

Supplement: S9 Fig — We find that increasing the number of nodes increases the number of interlocks. (TIF) [file pcbi.1010252.s009.tif]
