# Supplementary material for: Modeling cell biological features of meiotic chromosome pairing to study interlock resolution
Source: PLoS Comput Biol. 2022 Jun 13;18(6):e1010252. doi: 10.1371/journal.pcbi.1010252 (PMC9232156; doi:10.1371/journal.pcbi.1010252)
Supplement: Attachment — Submitted filename: Response to Reviewers.docx [file pcbi.1010252.s010.docx]

**Response to Reviewers**

We are greatly appreciative of the suggestions made by the reviewers and we have addressed and incorporated all of their comments. We feel that the manuscript has improved substantially with their suggestions. We addressed each of their concerns below:

**Reviewer 1**

**1. Number the equations.**

We have numbered the equations in the paper.

**2. The equations' description is incredibly sloppy, bordering on disrespect to the reader... What are zeta, m, etc? What is the physics behind the equations? m can't be mass , can it?? etc etc**

We apologize for not being as clear as possible. A definition has been given to each symbol in the methods, and we have also added sentences after technical portions that clarify the physics being simulated:

For example: *“Together, these equations simulate polymers undergoing Brownian motion in a viscous implicit solvent while including excluded volume interactions to prevent strand passage.”*

**3. Are the nodes of chromosomes jump between the lattice points or move in 3D continuous space?**

The nodes are free to move in 3D continuous space. The manuscript has been updated to make this fact clear.

We have added:

*“We note that this initial packing on a lattice is simply to ensure that nodes do not begin the simulation overlapping. During all subsequent simulation and equilibration steps the nodes are free to move in 3D continuous space.”*

**4. What is the 'indenter'?**

An indenter is a built-in function in LAMMPS whose purpose is to act as a constraining wall within a simulation. It is a force that acts on nodes close to the wall. In our case the indenter is spherical and acts to keep the nodes constrained inside a nucleus-like spherical region. We have updated the manuscript to make this clearer.

We have added the sentence: *“An indenter is a built-in function in LAMMPS whose purpose is to act as a constraining wall (in our case a spherical wall) within a simulation.”*

**5. Why is the spring force proportional to the square of the deformation? (why not use Hookean linear spring)? Why is the spring constant in pN/nm then?**

There was an error in the equation describing the spring force. It is in fact a hookean linear spring. It was previously described as a force when it should actually have been written as the derivative of a potential. We have corrected the problem in the manuscript.

**6. Why ten million steps? What does it correspond to in real time?**

We chose to simulate for ten million timesteps because it is the timescale by which our simulated cells have achieved a significant level of pairing. This corresponds to about ~1.4 hours in real time, which is of the right order for pairing in budding yeast (hours).

We have updated the manuscript to reflect this information by including the sentence

*“Unless otherwise stated, all simulations run for ten million timesteps. This corresponds to about 1.4 hours in real time, which is of the right order for pairing in budding yeast (hours).”*

**7. Telomeres' velocity - how is it directed? randomly? complete randomization at every time step?**

*“The simulation is essentially divided into 2000 timestep chunks. Every chunk of the simulation, each telomeric node has a 25% chance of experiencing an active pull. For each telomere experiencing an active pull, a random direction along the surface of the sphere is chosen. A force is applied in that direction for the entire chunk of time (2000 timesteps).”*

We now include the entire above statement in the main text.

**8. There are some vague hints to what the meaning of the parameters is in "Damping Time & Temperature", but I still do not understand any of it, despite my education in Physics. Come on guys! Explain what you are doing.**

The damping time is the relaxation time for the momentum and has units of time. It sets the viscosity in the simulation (smaller damping time means larger viscosity). We chose our damping time so that simulated measurements of the diffusion coefficient (595nm^2/s) approximated experimental measurements (500nm^2/s). We are now including this measurement as a supplementary Figure (S1).

Temperature appears in Equation 2 which determines the magnitude of the Langevin random force. It is used to set the magnitude of the thermal fluctuations.

We have added the following clarification to the manuscript:

“*ζ is the damping time that appears in Eq. 2 & Eq. 3. It sets the viscosity in the simulation (smaller damping time means larger viscosity).”*

**9. Re-write the Methods completely keeping in mind: say you want a student use your method. Explain to a student who never heard it before…**

Throughout the process of responding to these reviews we have added many clarifications, including a definition for each symbol that appears in each equation. We have also tried to break up technical portions with sentences that clarify the biology/physics being simulated. We think these changes make it much more understandable to the reader.

For example, the following sentence has been added to clarify the initial conditions that the initialization steps achieve:

“*These initialization/equilibration steps give us a starting configuration that represents two pairs (four chromosomes total) of homologous chromosomes inside of a nucleus with random relative orientations.”*

**10. Intro: not exactly clear what 'interlock' is; it is hard to understand from fig 1d how do telomeres' movements 'resolve interlocks'**

We have made small changes to figure 1d. We show that the RTMs can break apart homologous associations. This hopefully makes it clearer that the RTMs could help remove interlocks by breaking apart problematic pairing interactions. We have also added the following line to the figure caption.

*“(D) chromosome movement and two proposed roles: To increase collisions between homologous loci, or to resolve interlocks by breaking apart problematic pairing interactions.”*

We have also defined what an interlock is when the term first appears in the introduction as:

*“Interlocks are topological constraints that prevent pairing completion; they occur when a chromosome becomes trapped between another pair of chromosomes that have at least partially paired on each side.”*

**11. Fig 3b - speed of pairing may be statistically different in WT and mutants, but practically, the difference is insignificant, no? I see ~ 15% difference, Is that right? What kind of difference do people see experimentally? Probably much greater?**

In fact, the differences in pairing speed and extent that we see in our simulations are of the same order as those seen in actual mutants.

In [Lui 2013, Figure 5a] they measure and compare homolog pairing kinetics in WT and ndj1Δ cells. They do this by tagging a single locus on each homologous chromosome and measuring the fraction of time the spots appear colocalized.

Based on visual inspection of their figure, WT reaches a plateau that is approximately 10% higher than ndj1Δ, which is comparable to the difference we see (also approximately 10%).

Comparing the time it takes to reach half max: in their experimental study they see that WT cells reach half max approximately ~ 15% faster than ndj1Δ cells. This is comparable to our result, where WT cells reach half max approximately ~19% faster than ndj1Δ cells.

We thank the reviewer for pushing us to incorporate this comparison with experimental analysis in mutants. We have added the following to the manuscript:

“*In [32] the homolog pairing kinetics were experimentally compared in WT and ndj1Δ cells. This was accomplished by tagging a single locus on each homologous chromosome and measuring the fraction of time the spots appear colocalized. Based on visual inspection of their figure, WT reaches a plateau that is approximately 10% higher than ndj1Δ, which is comparable to the difference we see* ***(Fig 3B)*** *which is also approximately 10%.  In the same experimental study, it was found that WT cells reach half maximum pairing approximately ~ 15% faster than ndj1Δ cells, which is comparable to our result. Looking at our data in* ***(Fig 3B)****, our simulated WT cells reach half maximum approximately ~19% faster than our simulated ndj1Δ cells.”*

**Reviewer 2**

**1) The study is purely computational. While it is not a problem per se, it would really enhance the impact of the work if some quantitative or qualitative comparison is done with quantitative experimental data.**

Unfortunately, there is not a lot of data on pairing kinetics, or interlock resolution, but we have been able to make a simple comparison with published data in which quantitative information is provided for mutant versus wild-type pairing rates. Interestingly, the differences in pairing speed and extent that we see in our simulations are of the same order as those seen in actual mutants.

In [Lui 2013, Figure 5a] they measure and compare homolog pairing kinetics in WT and ndj1Δ cells. They do this by tagging a single locus on each homologous chromosome and measuring the fraction of time the spots appear colocalized.

Based on visual inspection of their figure, WT reaches a plateau that is approximately 10% higher than ndj1 Δ, which is comparable to the difference we see (also approximately 10%).

Comparing the time it takes to reach half max: in their experimental study they see that WT cells reach half max approximately ~ 15% faster than ndj1Δ cells. This is comparable to our result, where WT cells reach half max approximately ~19% faster than ndj1Δ cells.

We thank the reviewer for pushing us to incorporate this comparison with experimental analysis in mutants. We have added the following to the manuscript:

“*In [32] the homolog pairing kinetics were experimentally compared in WT and ndj1Δ cells. This was accomplished by tagging a single locus on each homologous chromosome and measuring the fraction of time the spots appear colocalized. Based on visual inspection of their figure, WT reaches a plateau that is approximately 10% higher than ndj1Δ, which is comparable to the difference we see* ***(Fig 3B)*** *which is also approximately 10%.  In the same experimental study, it was found that WT cells reach half maximum pairing approximately ~ 15% faster than ndj1Δ cells, which is comparable to our result. Looking at our data in* ***(Fig 3B)****, our simulated WT cells reach half maximum approximately ~19% faster than our simulated ndj1Δ cells.* "

**2) It is not very clear what the first 3 parts of the Results section (on pairing dynamics) bring compared to the previous works performed by the authors. Conclusions seems rather similar.**

While it is true that many of the conclusion of these results are similar to our previous work, it is important to note that our prior modeling work assumed a "phantom polymer" model for chromosomes, in that strands were able to freely pass through each other. The inclusion of excluded volume in this new model to prevent strand passage and allow for the study of interlocks changes the underlying physics, so that it was important to explicitly retest these ideas that we had previously addressed, with our more realistic model. But the main point was to focus on the generation and resolution of interlocks, something that our previous work could not address. We have updated our paper title to more correctly reflect the most important contributions of this paper (the study of interlocks).

**3) The main originality came from the study of interlocks. So, it might be nice to refer to the most original results in the title of the paper. Moreover, the term ‘interlock’ is directly used in the introduction and in the rest of the paper without precisely defining what is an ‘interlock’ (same for open or closed interlocks).**

We have updated our paper title to more correctly reflect the most important contributions of this paper (namely the study of interlocks). We have also edited the main text to include explicit definitions when these terms are first introduced:

*“Interlocks are topological constraints that prevent pairing completion; they occur when a chromosome becomes trapped between another pair of chromosomes that have at least partially paired on each side.”*

*“Open interlocks occur when one or both homologous chromosomes become trapped between another pair of homologous chromosomes and thus impede the pairing of a single pair of homologous chromosomes. Closed interlocks occur when the strands of two pairs of homologous chromosomes become trapped in a chain-like structure which prevents both pairs of homologous chromosomes from fully pairing.”*

**4) Overall, I found the paper very descriptive (with a nice analysis of the effects of many parameters & mechanisms) and it will gain in impact if more insights into the physics of the system were given. For example, why WT, ndj1Delta, csm4Delta have similar dynamics in Fig.3A but a different in Fig.3B? why ndj1Delta is more efficient than csm4Delta (Fig.3B)? what is the role of the interplay between zippering and inter-chromosome dynamics?**

To answer the first question we looked at time-course data for individual simulation runs in both cases (Figures S2 & S3). We see that in the case that every node can pair progressive zippering dominates the pairing behavior and eliminates differences between individual strains. In the case that pairing sites are spread out, progressive zippering is much weaker which leads to stronger differences between individual strains (& individual runs). We have added this information to the main text:

*“To better understand the differences between these two scenarios we looked at the time course data for individual simulation runs* ***(Fig S2)*** *&* ***(Fig S3****). We see that in the case that every node can pair progressive zippering dominates the pairing behavior and eliminates differences between individual strains. In the case that pairing sites are spread out, progressive zippering is much weaker which leads to stronger differences between individual strains (& individual runs).”*

As for the second question, using the data in Figure S2, we see that *csm4Δ* cells typically have a much slower ascent towards full pairing. *ndj1*Δ seems better able to take advantage of progressive zippering effects than *csm4Δ.* In the discussion section we argue that this is because the telomeric ends of the *csm4Δ* strain are highly constrained on the nuclear envelope. While both strains achieve the goal of disabling the RTMs, the way they achieve this effect is very different. In *ndj1Δ,* the ends come off the nuclear envelope, while in *csm4Δ* they remain attached. We have added the following statement to the discussion section:

*“To this effect, we see in* ***(Fig S3)*** *that csm4Δ cells typically have a much slower ascent towards full pairing than ndj1Δ. Indeed, ndj1Δ seems better able to take advantage of progressive zippering effects compared to csm4Δ.”*

**5) The interlock statistics and dynamics is likely to depend strongly on the initial configurations of the polymers. This is an important point that need to be studied. Experimentally, what is the know initial organization? Rabl?**

In yeast meiosis, the Rabl orientation falls apart before the onset of meiosis and is replaced by a bouquet configuration. We tested whether the nuclear bouquet led to a difference in the number of cells with interlocks and did not see a significant difference [Fig 8a].

**6) The section on interlock resolution would be really improved if some quantifications can be done. For example, estimation of the timescales of the different steps might be helpful.**

This is an excellent suggestion. To estimate the timescales of the different interlock resolution steps, we visually analyzed the trajectories of fifteen interlock resolution events using the software program OVITO.

For each interlock resolution event we noted the time it took to complete the three steps in interlock resolution: 1) Time to interlock migration, 2) Time to telomeric unpairing, and 3) Time to diffusive unwinding. While we note that there is some ambiguity in calling each of these steps visually, we tried our best to adhere to these rules when picking the timestep at which each of these events occurs.

1) Time to interlock migration: This is the timestep immediately preceding the interlock resolution event in which the interlock first gets within 10 nodes of the polymer endpoints.

2) Time to telomeric unpairing: Following interlock migration, this is the time it takes for the telomeric regions of the involved polymers to fully unpair.

3) Time to diffusive unwinding: Following telomeric unpairing, this is the time it takes for both pairs of homologous chromosomes to appear disentangled from each other such that a recurrence of an interlock is unlikely.

We display these results as a table in the supporting information (S5). We find that interlock migration is the limiting step in this process, taking an average of ~2850 seconds to complete, followed by diffusive unwinding at ~720 seconds, and telomeric unpairing at ~270 seconds.

We have added the following to the manuscript:

In Methods:

*“Timescales of interlock resolution steps: To estimate the timescales of the different interlock resolution steps, we visually analyzed the trajectories of fifteen interlock resolution events using the software program OVITO [Stukowski 2009]. For each interlock resolution event, we noted the time it took to complete the three steps in interlock resolution. While we note that there is some ambiguity in calling each of these steps visually, we tried our best to adhere to these rules when picking the timestep at which each of these events occurs. Time to interlock migration: This is the timestep immediately preceding the interlock resolution event in which the interlock first gets within 10 nodes of the polymer endpoints. Time to telomeric unpairing: Following interlock migration, this is the time it takes for the telomeric regions of the involved polymers to fully unpair. Time to diffusive unwinding: Following telomeric unpairing, this is the time it takes for both pairs of homologous chromosomes to appear disentangled from each other such that a recurrence of an interlock is unlikely.”*

In results:

*“To estimate the timescales of the different interlock resolution steps, we visually analyzed the trajectories of fifteen interlock resolution events using the software program OVITO. For each interlock resolution event we noted the time it took to complete the three steps in interlock resolution: 1) Time to interlock migration, 2) Time to telomeric unpairing, and 3) Time to diffusive unwinding.*

*We display these results as a table in the supporting information* ***(Fig S4)****. We find that interlock migration is the limiting step in this process, taking an average of ~2850 seconds to complete, followed by diffusive unwinding at ~720 seconds, and telomeric unpairing at ~270 seconds.”*

**7)	The Methods section is sometimes unclear and confusing.**

a. ‘F’ are forces but the equations describe sometimes a force but also sometimes a potential

In LAMMPS, some forces are described as forces, while some are described as derivatives of potentials. We chose to remain consistent with the terminology used in the LAMMPS documentation, in the interests of helping others to make use of our approach within LAMMPS.

b. The best potential to avoid chain crossing is FENE while authors use spring+LJ

Our choice of potential was guided by considerations of numerical stability in light of the way we initialize the state of our system with hard constraints. We chose to use harmonic spring potential + LJ because the FENE bond has a discontinuity at the maximum spring extension length that allows the energy to go to infinity. This discontinuity creates problems when “forcing” initial polymer configurations. For example: Our simulations require the telomeric nodes to be on the surface of the constraining sphere (the nuclear surface). Before t=0, the telomeric nodes are forced to this spherical surface by the constraint algorithm. If we used a FENE bond during this process the simulation would become unstable. Early in the project we decided to use spring+LJ simply due to its numerical stability.

c. The construction of the initial configurations should be better described (are all the polymers initialize simultaneously? what are their relative orientations? How the constraints for telomeres are managed?...)

At t=0 the polymers are randomly oriented inside the sphere after having gone through an equilibration to reach that random state.

We have added the following statement to the manuscript:

“*These initialization/equilibration steps give us a starting configuration that represents two pairs (four chromosomes total) of homologous chromosomes with random relative orientations, confined inside of a nuclear sphere.”*

The constraints on the telomeres are managed by a built-in Lammps command called **fix nve/manifold/rattle.** This algorithm is based on a paper that we have cited (Paquay 2016) and essentially uses the method of lagrange multipliers to ensure that the net force on each telomere is always perfectly perpendicular to the spherical surface that represents the nuclear envelope.

We have updated the manuscript to more clearly reflect this information.

*“To keep the ends of the chromosomes confined to the nuclear surface, we use the constraint algorithm described in [Paquay 2016], which ensures that the net force on each telomeric node is always perpendicular to the surface of the constraining sphere. This constraint is managed by a built-in LAMMPS function called nve/manifold/rattle.”*

d. How the polymers are confined inside a sphere is not clear. Is F (L113) a force? A potential? What is an indenter?

An indenter is a built-in function in LAMMPS whose purpose is to act as a constraining wall within a simulation. It is a force that acts on nodes close to the wall. In our case the indenter is spherical and acts to keep the nodes constrained inside a nucleus-like spherical region.

We have added a sentence in the methods section to make this more clear.

“*An indenter is a built-in function in LAMMPS whose purpose is to act as a constraining wall (in our case a spherical wall) within a simulation.”*

e. How the persistent forces on telomeres and the bouquet (size of the bouquet ?) are implemented?

The bouquet is implemented as a constant acceleration (force/mass) that drives the telomeres to a singular point on the nuclear surface. We note that since all nodes have the same mass this acceleration is essentially a constant force. The magnitude of this force can be adjusted to change the size of the bouquet (larger force results in a tighter bouquet).

We will update the manuscript to more clearly state this information.

*“For simulations involving a meiotic bouquet, an additional acceleration is exerted on the telomeric nodes that tend to keep these nodes in a subregion of the nuclear surface. The bouquet is modeled as a weak constant acceleration (force/mass) that drives the nodes toward a single point on the nuclear surface. The size of the bouquet can be modulated by adjusting the magnitude of this acceleration.”*

f. Motivations behind the choices of spring constants are not given.

The spring constant was set high enough to prevent strand passage. We have updated the manuscript with this information.

*“Our primary concern when choosing a spring constant is to set it high enough to prevent strand passage.”*

g. Regarding time mapping: authors compare the predicted MSD to experimental observations but do not show a figure. Moreover, for a polymer, MSD scales as t^(1/2) so the unit of effective diffusion coefficient is in [length]^2/[time]^(1/2) and not [length]^2/[time] as for normal diffusion. More recent estimation of chromatin dynamics in yeast may be found in Hajjoul et al, Genome Research 2013.

This is a very interesting point. In standard texts on polymer dynamics [Doi 1978] we see that for short times the monomer mean square displacement does indeed scale as t^(1/2), however for long times, the center of mass motion of the chain dominates, and the monomer MSD scales as t^(1). In the paper described above [Hajjoul 2013], they are measuring the MSD in the short time limit, so for that study, a scaling of t^(1/2) was the appropriate relation. In our case, the timescale of meiotic pairing is very long (hours) and requires motion of the entire polymer chain, so we think in this case it is appropriate to look in the long-time limit.

We have now included our measurement of the diffusion coefficient as a supplementary figure **(Fig S1)** and cited the figure in the paper. We do indeed see sub-diffusive scaling at short times, with a transition that occurs at an MSD of nb^2, where n is the number of nodes, and b is the kuhn length, exactly as predicted by theory.

Doi, M.; Edwards, S. Dynamics of concentrated polymer systems. Part 1. Brownian motion in the equilibrium state. J. Chem. Soc., Faraday Trans. 2 1978 , 74, 1789 −1801.

**8) Entanglements and knots have been discussed also a lot in the context of interphasic chromosomes (eg, crumpled polymers). Might be interesting to compare with authors’ results.**

This is a good point. While we feel that a detailed discussion of interphase chromosome entanglement is beyond the scope of our paper, we have added the following sentence to the manuscript in order to provide some references that would provide a starting point to allow a reader to explore this literature:

*“While the term interlocks is usually used in the context of Meiosis, recent work has raised the idea that interphasic chromosomes may also be partially knotted [20–22].”*

**9) Authors rightly associate the formation of interlocks to entanglement, it might be interesting to discuss this a bit more (what is the entanglement length of the simulated polymer?) Long polymers are more sensitive to entanglement, what authors’ conclusions depend on the size of the polymer they choose?**

This is an excellent question. To address this question, we used primitive path analysis to count the number of entanglements along our polymer chains. Primitive path analysis [ref] is a computational method in which you force a polymer to condense to its primitive path. The primitive path can be defined as the shortest path through all of the entanglements.

For an unentangled polymer the primitive path will be the shortest straight line that connects both polymer endpoints. For an entangled polymer however, the primitive path will have “kinks” at places of entanglement due to excluded volume interactions. These “kinks” can be counted and directly represent the number of entanglements along a polymer chain.

We ran the primitive path algorithm on 15 simulations. For each simulation we equilibrate the four polymers as usual. At the end of the equilibration, we designate one of the four polymers as the polymer of interest (the PPA algorithm requires you to choose a polymer of interest), run PPA algorithm on that polymer and then manually count the number of entanglements along that polymer chain.

We did this for three different nuclear radii and find that our polymers contain between 0-4 entanglements per polymer, with an increasing number of entanglements as you decrease the nuclear radius. Increasing the degree of confinement thus seems to increase the degree of entanglement.

Another way to modulate the degree of entanglement is to increase the number of nodes per polymer chain. To this effect, we ran pairing simulations at three polymer lengths (N=80, N=100, and N=120) and evaluated them at the end for the existence of interlocks **(Fig S6)**. We find that the number of interlocks increases as you increase the number of nodes per polymer chain.

From these two measurements we can conclude that as the degree of entanglement increases, the number of interlocks will also increase, but we do not think this would qualitatively change any of our conclusions.

We have added the following paragraphs to the results section of the main text:

*“The number of interlocks is likely to depend strongly on the degree of entanglement. There are two ways to easily modulate the degree of entanglement in our model 1) change the volume of confinement, and 2) change the number of nodes per polymer chain.*

*First, we sought to quantify the number of entanglements per polymer chain for three different nuclear radii. To do this, we used primitive path analysis as discussed in the methods section. We find that our polymers contain between 0-4 entanglements per polymer, with an increasing number of entanglements as you decrease the nuclear radius* ***(Fig S5)****. In* ***(Fig 8b)*** *we also showed that increasing the nuclear radius decreases the number of interlocks. Together these results support the idea that increasing the degree of entanglement leads to more interlocks.*

*Another way to modulate the degree of entanglement is to modulate the number of nodes per polymer chain, as longer polymers will be more highly entangled [de Gennes 1971]. To test whether using longer polymers leads to more interlocks, we ran pairing simulations at three polymer lengths (N=80, N=100, and N=120 nodes per chain) and evaluated them at the end for the existence of interlocks* ***(Fig S6)****. We find that the number of interlocks increases as you increase the number of nodes per polymer chain, which supports the idea that the number of interlocks depends on the degree of entanglement.”*

**10) References are cited as (Name Date) format but in the reference list, they are numbered.**

We have now used a consistent citation scheme throughout.

**11)	L93: Leonard-> Lennard**

thank you for noticing this typo, which we have now fixed.

**12)	L106 : ‘matched theoretical predictions’: which one ?**

We did a quick check to make sure that if we turn off excluded volume we get the right radius of gyration for a rouse polymer <R> = l. The theoretical prediction for=100, and l = 100 is 408.2 nm. Our measured radius of gyration is 396.7 nm with a standard deviation of 98 nm. This was simply to ensure that the polymers weren’t still compacted from their initialization on a compact lattice. The purpose of the lattice initialization was to ensure that 1) the nodes didn’t begin the simulation overlapping, which would cause simulation instability. 2) Ensure the polymers were initialized inside the nuclear sphere. We checked the radius of gyration to make sure this initialization choice wasn’t affecting future measurements (if for example, the polymers were still partially compacted we would expect to get a smaller than predicted radius of gyration).

**13)	For some more recent estimations of the chromatin persistence length in yeast, authors may cite [Arbona et al, Genome Biology 2017; Socol et al , NAR, 2019].**

We now cite these papers when discussing the persistence length of chromatin. In [Arbona 2017] they estimate that the persistence length of chromatin is 52 nm- 87 nm. In [Socol 2019] they infer the kuhn length from simulations of the nucleosome crystal structure and determine that the kuhn length is between 95-120 nm, which corresponds to a persistence length of 47.5nm - 60nm. Both of these measurements are close to the persistence length used in our model (50 nm).

**14)	L335: “it has been postulated that….”, any reference?**

We have added references for this statement. [Conrad 2008, Koszul 2008, Martinez-Garcia 2018]

**15)	L340: how the initial open & closed interlock configurations were chosen?**

We have added the following statement to the main text:

*“The initial open and closed configurations were chosen so that the interlock was near the center of the polymers. These initial configurations were then randomized via an equilibration step that did not allow the bond topology to change (to prevent the interlock from resolving before t=0). We have updated the manuscript to reflect this information.”*

**16)	L359: ‘slightly slower pairing’: the slowdown is as for ndj1Delta**

We see a progressive slowdown as you detach the ends. With both ends attached pairing occurs the fastest, followed by with a single end attached, followed by with both ends detached.

**17)	Panel 9D is not cited in the text**

Thank you, we have made sure to correctly cite this figure in the text.

**18)	L395-401: maybe delocalized to the Method section**

We have moved these paragraphs to the methods section.

**19)	In the Discussion section, authors should discuss more clearly the contradictions of their results with results from Martinez-Garcia et al, 2018**

We have added the following paragraphs to the discussion section:

*“In Martinez-Garcia et al, 2018, they show that an RTM deficient A. Thaliana mutant, nup136Δ has more interlocks than the wild-type cells. Importantly, this result seems to contradict the conclusions presented in the current study. Indeed, our simulated RTM deficient yeast strains contain less interlocks than in the WT cells. We would like to speculate on a possibility for this apparent contradiction.*

*First, we note there are well established differences between the canonically studied RTM deficient budding yeast strains csm4 Δ and ndj1 Δ. Experimentally, it is well established that csm4 Δ strain is more deleterious than ndj1 Δ, both in terms of time it takes to enter anaphase, as well as spore viability. The simulation study presented here supports these results: we see that csm4 Δ is both slower at pairing and has more interlocks than ndj1 Δ. Importantly, these differences arise from a key difference between these two mutants (which we built into our model): the behavior of the telomeres. In ndj1 Δ the telomeres detach from the nuclear envelope, while in csm4 Δ they remain attached. Understanding the behavior of the telomeres is thus key to understanding the physical effect of these mutations. While both achieve the goal of turning off the RTMs, they do it in two very different ways which results in very different physical situations.*

*To our knowledge, there is no published study that carefully examines the behavior of the telomeres in nup136Δ. Additionally, nup136 Δ has been shown to alter the nuclear morphology [Tamura 2010], going from highly elongated in WT cells, to highly spherical in the mutant strain, an effect not seen in either ndj1 Δ or csm4 Δ. There is thus no true contradiction. Each RTM deficient strain is unique and careful attention to detail is required to model specific strains. There is unfortunately currently not enough experimental data on the velocity and localization of the telomeres in nup136Δ to model this strain. Perhaps a future version of the model will incorporate future data to better understand*

*interlock resolution in this strain.”*

**20)	L554-L558: many polymer models of chromosome organization are using similar formalism, so I am not sure that this paragraph is really useful.**

Thank you for your suggestion, we have removed the paragraph.
