# Supplementary material for: Modeling cell biological features of meiotic chromosome pairing to study interlock resolution
Source: PLoS Comput Biol. 2022 Jun 13;18(6):e1010252. doi: 10.1371/journal.pcbi.1010252 (PMC9232156; doi:10.1371/journal.pcbi.1010252)
Supplement: Attachment — Submitted filename: ReviewerResponses.docx [file pcbi.1010252.s011.docx]

May 24, 2022

We are greatly appreciative of all the additional suggestions made by the reviewers and we have addressed and incorporated all of their comments. We feel that the manuscript has improved substantially with their insightful suggestions. We addressed each of their concerns below:

**Reviewer 2**

**1. The expression in Eq.2 is the standard deviation of the normal distribution used to simulate the stochastic force, it is not the expression of the force itself.**

Thank you for pointing this out. We have added the following statement to the manuscript to clarify:

*“F_r is the root mean square magnitude of the Langevin random force due to interactions with the solvent”*

**2. in Eq 4, r -> r_{i,i+1}**

We have added the indices as suggested.

**3. in Eq.5, r-> r_{i,j} and \in -> \epsilon**

We have added the indices, and used the correct symbol for epsilon.

**4. in Eq.2, K_b-> k_b**

Thank you for catching this typographical error, we have now corrected it.

**5. Eq.6: K is different from Eq.4 I guess, please use different notation, same for 'r'.**

We have added subscripts to the two different k parameters in the two equations, and we now use r for equation 4 and s for equation 6. We have also added the domain of the indenter force to equation 6.

**6. For the RTM, what is the shape and amplitude of the active pulling force? same for the constant acceleration for the bouquet ?**

For both the RTM and bouquet, the added force is a straight vector, however, when combined with the constraint algorithm it produces curved motion on the nuclear surface. For the RTM, the magnitude of the added force is 0.312 pN, in the case of the bouquet, the magnitude of the added force (since all nodes have the same mass) is 0.187 pN. We have now added this information to the table of values in Figure 2.

We have also made the following modifications to the manuscript:

*For each telomere experiencing an active pull, a random straight-line direction is chosen.*

*For simulations involving a meiotic bouquet, an additional straight-line acceleration is exerted on the telomeric nodes*

**7. To model the pairing, two nodes are paired with a harmonic spring (what is the value of the spring constant ?**

The value of that spring constant is 0.0285 pN/nm. We have added this information to the table of values in Figure 2b.

**8. How the pairing parameters (binding/unbinding rate) were chosen ?**

The pairing parameters were chosen so that our simulated chromosomes reach a level of pairing that approximately matches experimental data. In [Newman 2022] experiments were reported in which a single homologous locus was tagged on two homologous chromosomes and its pairing status tracked over time. That study found that the two spots reach a level of colocalization of approximately ~60%. In our simulations, parameters were chosen so that approximately ~55% of the pairing nodes are paired after a full run. We have added the following to the manuscript:

“*In [6], a single locus was tagged on both homologous chromosomes and tracked over time. In that study, the spots corresponding to homologous loci reached a level of colocalization of approximately ~60%. Our pairing/unpairing parameters were chosen so that approximately ~55% of the pairing nodes are paired after a full run. This corresponded to a pairing probability of 85% (if within the cutoff radius), and an unpairing probability of 0.00125%*

Newman TAC, Beltran B, McGehee JM, Elnatan D, Cahoon CK, Paddy MR, et al. Diffusion and distal linkages govern interchromosomal dynamics during meiotic prophase. Proceedings of the National Academy of Sciences. 2022 Mar 22;119(12):e2115883119.

**9. Authors claimed they adjust the pulling forces to have speed of 0.6 um/s. how was it done? any Sup Fig to illustrate that fit?**

We have added a supplementary figure (S2) that shows the velocity of a single telomere through time in WT cells. We have also referenced this figure within the manuscript. Our approach for adjusting the pulling forces was to set the magnitude of the active forces and the probability of receiving an active pull such that the speed distribution resembled experimental descriptions in terms of both the peak speeds of motion and the timescale of active movements and intervening dwell times.

Experimentally, motion of the telomeres is characterized by periods of active pulling during which the telomeres reach max speeds of 0.6um/s (in most cells). These active pulling periods last on the order of seconds. In between pulls there are dwell times in which telomeres are relatively immobile, and these periods also last on the order of seconds. Our simulated active pulls capture this behavior: The telomere pulling events last for ~1.0 seconds, with dwell times that also last on the order of seconds.

**10. It is unclear how the telomere tethering at the nuclear periphery is forced at the initiation stage (when the initial configuration is built).**

Let N=1 be the first node on the chain, and N=2 be the second node, etc. The N=1 node technically starts out near the nuclear envelope, but the next node (N=2) is on a cubic lattice far away (far compared to the resting length of the spring that connects them.) During the first few timesteps of the initialization procedure these two nodes are being brought together near the nuclear envelope by a combination of the constraint algorithm, and the harmonic spring that connects them. The energies associated with this procedure are high enough that the constraint algorithm would normally fail to constrain the N=1 node to the nuclear envelope. The trick here is to “force” the N=1 node to stay on nuclear envelope, which we achieve by capping the distance any node can move in a single time step to a distance 1/10 the size of a node. We find this allows the constraint algorithm to keep the N=1 nodes on the nuclear envelope until the energies stabilize (until the N=2 node is near the nuclear envelope, or to be more precise, until the N=2 node is within one resting spring length of the N=1 node.)

We have added the following line to the manuscript

*All nodes except for the terminal nodes start on this lattice. The terminal nodes begin on the nuclear membrane.*

**11. It is not obvious to see on Fig.S2 & S3 that zippering is dominant or weaker. Maybe a representation of individual trajectories as kymographs (as in Marshall & Fung, 2016) might be more explicit.**
We have included the requested kymographs as two figures in the supporting information. We agree that this helps highlight the differences in zippering between the two cases. In the case that every node can pair we see a sudden near vertical shift to blue, suggesting that zippering is the dominant pairing behavior. In the case that pairing sites are spread out this shift to blue happens more gradually. We have also referenced the new figures in the main text.

After seeing these new plots, we also removed the following line from the discussion: “Indeed, *ndj1Δ* seems better able to take advantage of progressive zippering effects compared to *csm4Δ*.” Since it was not that clear from the new plots that a difference in progressive zippering is the reason for the differences between these two strains.

**12. Put Arabidopsis in italic.**

Thank you for catching this. We have corrected this in the manuscript.
